# New Insight into Potential Otoprotective Effects of Lactoferrin: Is It Paradoxically Ototoxic? An Experimental Investigation

**DOI:** 10.3390/audiolres16020040

**Published:** 2026-03-06

**Authors:** Ahmet Mutlu, Ayse Yasemin Gunduz, Burcu Bakici, Murat Erinc, Erdogan Bulut, Onur Ersoy, Serdal Celik, Dogan Cakan, Mahmut Tayyar Kalcioglu

**Affiliations:** 1 Department of Otorhinolaryngology, Faculty of Medicine, Istanbul Medeniyet University, Istanbul, Türkiye; 2 Department of Otorhinolaryngology, Göztepe Prof. Dr. Süleyman Yalçın City Hospital, Istanbul, Türkiye; 3 Department of Otorhinolaryngology, Mardin Training and Research Hospital, Mardin, Türkiye; 4 Department of Otorhinolaryngology, Çankırı State Hospital, Çankırı, Türkiye; 5 Department of Audiology, Faculty of Health Sciences, Istanbul Medeniyet University, Istanbul, Türkiye; 6 Department of Audiology, Faculty of Health Sciences, Trakya University, Edirne, Türkiye; 7 Mirko Tos Ear & Hearing Research Center, Edirne, Türkiye; 8 Department of Pathology Laboratory Techniques, Vocational School of Health Services, Trakya University, Edirne, Türkiye; 9 Department of Otorhinolaryngology, Cerrahpasa Faculty of Medicine, Istanbul University, Istanbul, Türkiye

**Keywords:** lactoferrin, cochlea, ototoxicity, hearing loss, Sprague-Dawley rat

## Abstract

To evaluate the potential ototoxic effects of lactoferrin on the inner ear using electrophysiological and histological methods. Methods: Thirty-two Sprague-Dawley rats (64 ears) were divided into four groups: control, saline, antiseptic solution (70% isopropyl alcohol + 2% chlorhexidine), and lactoferrin. Groups II–IV received three intratympanic injections. Auditory brainstem response (ABR) tests were performed at baseline, day 7, and day 21. Cochlear histology and VEGF immunoreactivity were assessed. Results: Baseline hearing was similar across groups. Post-treatment, Groups II and IV showed partial recovery at 8, 16, and 24 kHz, while Groups III and IV had worsening thresholds at higher frequencies. Histologically, Group IV’s cochlear structures remained largely intact. VEGF immunoreactivity was severe to moderate in Groups I, II, and IV, and weaker in Group III. Conclusions: Lactoferrin showed relative safety at lower frequencies but possible ototoxicity at higher frequencies. However, no significant structural damage was observed in cochlear tissues.

## 1. Introduction

Lactoferrin is a multifunctional mammalian glycoprotein with iron-binding capacity, which is naturally found in mucosal secretions such as saliva, tear, and mainly breast milk. This endogenous biomolecule is known to possess antimicrobial (antibacterial, antiviral, and antifungal), anti-inflammatory, and antioxidant properties, rendering the biological fluids a crucial element of the body’s defense mechanisms [1]. With these potent features, it holds important biological roles including regulation of inflammatory diseases by immune system enhancement and infection control by defensing against the pathogens [2,3,4]. In particular, antioxidant characteristics are of interest due to its potential to minimize cell damage through prevention of free radical formation [5].

Ototoxicity is referred to as disturbance of inner ear structures or functions by chemicals or pharmaceuticals. Sensory cells of the inner ear, particularly outer hair cells, are highly vulnerable to oxidative stress and chemical insults [6]. Some antibiotics such as aminoglycosides, some diuretics such as furosemide, some chemotherapeutic agents such as cisplatin, and several antiseptics have been demonstrated to be ototoxic with severe damage to cochlear structures [7,8].

Given the protective effects of lactoferrin, its utilization against various toxicities throughout the body has been a subject of interest and many investigations have taken place in the literature within this scope [9,10,11,12,13]. A very limited number of studies in the field of otology with lactoferrin have been conducted, confined to its use in infective otitis media and its therapeutic role in age-related hearing loss [14,15,16]. Moreover, there is not enough research to clearly demonstrate the effects of lactoferrin on sensitive otologic structures. Specifically, in addition to its potential protective effects, there is no research investigating whether it has an ototoxic effect.

The aim of this study was to investigate experimentally whether lactoferrin has ototoxic effects in the inner ear. In particular, the effects of lactoferrin were examined electrophysiologically on low-, medium-, and high-frequency hearing thresholds and histopathologically, and the mechanisms of the resulting effects were discussed.

## 2. Materials and Methods

### 2.1. Ethical Approval

The research was conducted after the approval of Istanbul Bezmialem Vakif University Laboratory Animals Local Ethics Committee on 26 April 2021 (Decision number: 2021/137) in accordance with Directive 2010/63/EU on the protection of animals used for scientific purposes.

### 2.2. Study Setting and Design

This prospective experimental study was set up as a randomized-controlled investigation and performed in the Laboratory of Experimental Animals of Istanbul Bezmialem Vakif University and Mirko TOS Ear and Hearing Research Center of Trakya University in Türkiye, respectively.

The study was designed to include a total of 32 animals (64 ears) after performing post hoc power analysis to assess sample size adequacy. All the subjects were Sprague-Dawley rats of male gender with a weight of 250–300 g and age of 9–10 weeks. As the estrous cycle could have an effect on electrophysiological responses during the tests, female rats were excluded. Under the environmental temperature of 21 ± 2 °C and 12 h light/12 h dark cycle, the animals were sheltered at four per cage with ad libitum supply of water (from the mains water of the city) and food (pellets of standard rodent chow diet) [17].

The rats were randomized into 4 groups of equal size, each group including 8 rats (16 ears): Group I was the blank control group where no intervention was made. Group II was the negative control group where 0.9% isotonic sodium chloride solution was injected. Group III was the positive control group where antiseptic solution of 70% isopropyl alcohol + 2% chlorhexidine (Dermol, Biorad, Istanbul, Türkiye) was injected. Finally, Group IV was the study group where lactoferrin solution at a concentration of 250 mg/mL (Human Recombinant Lactoferrin, Merck Millipore, Darmstadt, Germany) was injected. Each intervention group (Group II, III, and IV) received a 0.03 mL injection of the aforementioned substances three times, every 2 days.

All procedures were performed under general anesthesia induced intraperitoneally with 5 mg/kg ketamine hydrochloride and 5 mg/kg xylazine, with an additional one-third of the initial ketamine hydrochloride dose given intraperitoneally if the depth of general anesthesia diminished. Microscopic ear examination using a suitable size ear speculum was performed for each ear as the initial step after the induction of general anesthesia. Factors occluding the ear canal, such as ear cerumen or fluid, were cleaned to have a clear visual access to the tympanic membrane. The tympanic membranes were examined for perforation or effusion for exclusion. Since none of the 64 ears revealed abnormal tympanic membrane examination, no exclusion was made. An intratympanic injection using a 27 Gauge dental needle through posteroinferior quadrant of the tympanic membrane was performed delicately not to damage the malleus and the ossicular chain. During the injection, 0.03 mL volume of injected material was observed behind the tympanic membrane to fill the middle ear cavity of the rat.

During the first general anesthesia, basal hearing levels of each ear were recorded with auditory brainstem response (ABR) after the initial ear examination under microscope. After the three intratympanic injections which are 2 days apart from one another were completed, early-phase hearing levels were measured with ABR 2 days apart from the third injection, which is the final injection. Two weeks after the early-phase ABR measurements, late-phase hearing levels were measured with ABR. That way, early- and late-phase hearing levels were obtained on the 7th and the 21st days after the first ABR measurement determining the basal hearing level, respectively [18]. While background noise levels were not objectively measured with a sound level meter, all ABR testing was conducted in a quiet clinical environment.

After performing the last ABR test on day 21, sacrification of the rats were performed with cervical dislocation and decapitation under guillotine. Both temporal bones of each rat were removed by surgical dissection and skeletonized by extraction of the soft tissues, and the bullar bones were opened with a hole to allow for the fixative solution to infuse. The temporal bones were preserved in the 10% formalin solution for one week at a temperature of +4 °C and one more week at room temperature for fixation before the histological investigation of the cochlea.

### 2.3. Electrophysiological Measurements

Electrophysiological assessments were performed using the ABR test to determine hearing thresholds of the study animals. All procedures were conducted under general anesthesia to minimize movement artifacts and ensure animal comfort and researcher safety. Anesthesia was induced by intraperitoneally administered 5 mg/kg ketamine hydrochloride and 5 mg/kg xylazine, with additional ketamine administered as needed to maintain adequate sedation. During the procedure, rats were covered with cotton blankets to maintain their body temperature to prevent hypothermia.

ABR recordings were conducted using Intelligent Hearing Systems (IHS) Smart EP version 5.10 (IHS^®^, Miami, FL, USA) and IHS high-frequency transducers (IHS, Miami, FL, USA). The transducers were connected to insert earphones, and a probe appropriately sized for the rats’ external ear canals was used to ensure optimal sound delivery. Recordings of the responses were performed by needle electrodes placed subdermally at the vertex (positive), ipsilateral mastoid (negative), and contralateral mastoid (ground). Impedance levels were maintained below 1 Ω to ensure signal quality and minimize noise interference. These transducers deliver the stimulus directly into the rat’s ear canal via a slim tube with a probe at the tip, without any intermediate device. The equipment is calibrated annually by the authorized company.

A tone burst stimulus of 1000 μs duration was delivered with a 37.1/s repetition rate using the Blackman envelope. Measurements were obtained at 8, 16, 24, and 32 kHz to assess frequency-specific hearing thresholds. The recordings were filtered through a 30 to 3000 Hz band-pass filter, and each response was averaged over 1024 sweeps to enhance waveform clarity.

Hearing threshold determination began at 80 dB sound pressure level (SPL), followed by 10 dB SPL decrements. As the threshold level was approached, smaller steps of 5 dB SPL were employed to improve precision. The hearing threshold was defined as the lowest sound intensity level at which a repeatable wave II, the most prominent ABR waveform in rats, was identified. To confirm the reliability of the results, responses at the determined threshold were obtained with tests repeated at least twice to ensure reproducibility. The hearing threshold for normal hearing was accepted as 20 dB SPL [18,19].

### 2.4. Histological Investigation

After fixation, the temporal bones harboring the cochlear samples were incubated with phosphate-buffered saline (PBS) to cleanse any remaining fixative residues. For decalcification, the temporal samples were immersed in a 0.1 M Na-EDTA (Sigma, Darmstadt, Germany) solution at pH 7.4 at room temperature for two weeks. This step ensured proper softening of the bony structures to facilitate precise sectioning. The tissues then underwent a dehydration process through a graded series of alcohol solutions. Following dehydration, the tissues were cleared using xylene to enhance tissue transparency and subsequently embedded in paraffin for sectioning. From the paraffin blocks, 5 μm thick sections were obtained and subsequently deparaffinized and rehydrated. The sections were then stained with hematoxylin–eosin (H-E) to enable histological evaluation. Microscopic examination was conducted using an Olympus BX51 (Tokyo, Japan) light microscope.

The organ of Corti (OC), with its outer hair cells (OHCs) and inner hair cells (IHCs), stria vascularis (SV), and spiral ganglion cells (SGs), were carefully evaluated for morphological changes. Degenerative alterations in these structures were assessed using a scoring system adapted from de Freitas et al. [20]. OHCs and IHCs were scored based on cytoplasmic features, cellular integrity, stereocilia arrangement, and nuclear condition, with a four-tier scoring system (0: Normal/No change, 1: Mild damage, 2: Moderate damage, 3: Severe damage). SGs were assessed and categorized by examiner’s visual assessment for vacuolization and nuclear degeneration (0: Normal/No change, 1: Mild change, 2: Moderate change, 3: Severe change). SV evaluation was based on SV thickness as well as marginal cell blebbing, vacuolization, and atrophy of intermediate and basal cells, with the same scoring scale applied (0: Normal/No change, 1: Mild change, 2: Moderate change, 3: Severe change) [21]. Notably, the mean thickness of the SV was measured using a computer-assisted image analysis program (ArgenitKameram 2.11.5.1, Istanbul, Türkiye) by performing 10 orthogonal measurements across the full length of the SV at each cochlear turn [22].

### 2.5. Hematoxylin–Eosin Staining Process

The histological sections obtained from 5 μm thick paraffin blocks were prepared for staining through a standardized deparaffinization and staining protocol. Initially, the sections were immersed in toluene for 30 min to remove the paraffin residues. Following this step, the samples were rehydrated by sequentially passing through a series of graded alcohol solutions in descending concentrations of 100%, 96%, 90%, and 70%, respectively; and subsequently immersed in distilled water to complete the rehydration process. For nuclear staining, the sections were incubated in Mayer’s hematoxylin solution (Merck Millipore, Darmstadt, Germany) for 10 min. The sections were then rinsed under running tap water for 10 min to enhance nuclear definition. For cytoplasmic staining, the sections were exposed to Eosin solution (Merck Millipore, Darmstadt, Germany) for 1 min, ensuring clear visualization of the cytoplasmic components. The dehydration process was performed by sequentially passing the sections through an ascending series of alcohol solutions with increasing concentrations of 70%, 90%, 96%, and 100%, respectively. Following dehydration, the sections were again treated with toluene for 30 min to enhance tissue transparency. Finally, the sections were permanently sealed with Entellan (Merck Millipore, Darmstadt, Germany) to preserve the stained tissues and ensure long-term sample integrity.

### 2.6. Immunohistochemistry Process

Immunohistochemical analysis to assess potential ototoxic effects and cochlear degeneration was performed with vascular endothelial growth factor (VEGF) immunoreactivity in cochlear tissues. VEGF expression was evaluated in key cochlear structures, including the SV, SL, SG, and OC, to assess their regenerative capacity in vasculature and tissue integrity.

Paraffin-embedded tissue sections with a thickness of 5 μm were put onto poly-L-lysine-coated slides, then the sections were incubated overnight at 56 °C to improve tissue adherence. Following incubation, deparaffinization was performed by immersing the sections in toluene for 30 min to remove residual paraffin. The sections were then passed through a series of graded alcohol solutions with decreasing concentrations of 100%, 96%, 90%, and 70%, respectively, to facilitate rehydration, followed by immersion in distilled water.

For antigen retrieval, the sections were incubated inside chalets with citrate buffer solution of pH 6.0 and heated in a microwave oven (Vestel 1550, Manisa, Türkiye) for 4 cycles of 5 min each. Following this, the slides were cooled to room temperature and subsequently washed three times for 5 min each in 0.01 M PBS of pH 7.2–7.4. To hinder endogenous peroxidase activity and minimize background staining, sections were treated with 3% hydrogen peroxide (H_2_O_2_) solution prepared in distilled water for 5 min and washed again with PBS three times for 5 min each. To block non-specific antibody binding, sections were incubated with 1% non-immune rabbit serum (Ultra V Block, TA-015-UB; LabVision, Fremont, CA, USA) for 5 min.

For VEGF detection, the sections were incubated with a rabbit polyclonal VEGF antibody (LS-C389419; LifeSpanBioSciences, Seattle, WA, USA) diluted at 1:200 in antibody dilution solution (Invitrogen, Carlsbad, CA, USA) for 1 h at room temperature. Negative control sections were not treated with primary antibody solution; instead, they were treated with PBS. Next, the sections were treated with a biotinylated secondary antibody targeting the strain from which the primary antibody was derived (Invitrogen, CA, USA) for 10 min. This was followed by incubation with horseradish peroxidase-streptavidin (HRP-streptavidin) (Invitrogen, CA, USA) for additional signal amplification.

For chromogenic staining, sections were incubated with 3,3′-diaminobenzidine tetrahydrochloride dihydrate (DAB) (Invitrogen, CA, USA) to visualize immunoreactive sites. Finally, the sections were counterstained with hematoxylin, dehydrated, and sealed with Entellan (Merck Millipore, Darmstadt, Germany) to ensure long-term preservation. The prepared slides were examined using an Olympus BX51 research microscope (Tokyo, Japan) for VEGF immunoreactivity.

VEGF immunoreactivity in the SV, SL, SG, and OC regions was evaluated using a semi-quantitative scoring system as follows: (-) No staining, (+) Weak staining, (++) Moderate staining, (+++) Strong staining. Additionally, the thickness of the SV was measured in all groups as an indicator of early ototoxicity. All histological and immunohistochemical evaluations were conducted by two independent investigators blinded to group of the specimens to ensure unbiased assessment.

### 2.7. Statistical Analysis

Statistical analysis was conducted using the SPSS for Mac v20 software (IBM, Armonk, NY, USA) with a significance level set at *p* < 0.05. To assess the normality of the ABR data distribution, the Shapiro–Wilk test was employed. Since the data did not exhibit a normal distribution, non-parametric tests were utilized for further analysis. The Kruskal–Wallis test was applied for multiple inter-group comparisons, while the Friedman test was used for intra-group comparisons. For analyses that revealed statistically significant differences, post hoc power analysis was conducted to evaluate the adequacy of the sample size and post hoc pairwise comparisons were conducted to identify the source of these differences and understand inter-group and intra-group variability.

## 3. Results

### 3.1. Electrophysiological Findings

ABR measurements for each frequency within the groups and among the groups were analyzed. The basal hearing levels of each group and the Group I hearing levels on different days across the study period did not show any difference, indicating that the rats had uniform hearing levels and the hearing of the rats was not influenced by the passing time (Table 1, Table 2, Table 3 and Table 4).

Inter-group comparisons of ABR measurements on day 7 and ABR measurements on day 21 yielded significant differences among the groups and pairwise comparisons were carried out (Table 1, Table 2, Table 3 and Table 4). At lower frequencies (8 and 16 kHz), Group II and IV hearing levels were similar as they did not differ statistically from each other in both early- and late-phase ABR tests (Table 1 and Table 2). However, at higher frequencies, Group IV hearing levels showed no significant difference from Group III, specifically, in early-phase ABR tests at 24 kHz, and with the progressive deterioration of the hearing in late-phase at 32 kHz (Table 3 and Table 4).

Importantly, the electrophysiological alterations were further substantiated by histological and immunohistochemical findings, which provided morphological evidence for the observed threshold shifts.

### 3.2. Histology and Immunohistochemistry Findings

**Figure 1:**
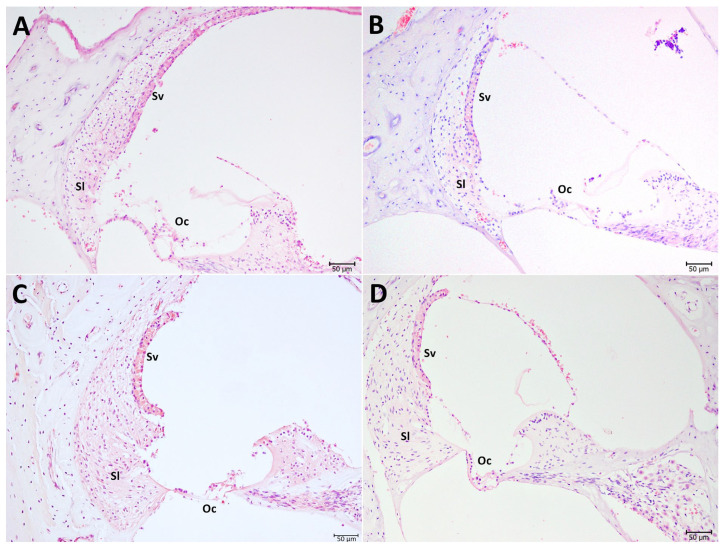
Hematoxylin–eosin (H-E) stain of cochlear structures. (**A**) Group I, blank control; (**B**) Group II, 0.9% isotonic sodium chloride; (**C**) Group III, antiseptic solution (70% isopropyl alcohol + 2% chlorhexidine solution); (**D**) Group IV, lactoferrin. Oc: Organ of Corti. Sl: Spiral ligament. Sg: Spiral ganglion. Sv: Stria vascularis.

Histopathological evaluation with H–E staining revealed preserved cochlear morphology in Groups I (control) and II (saline). In Group III (antiseptic solution), however, moderate degeneration according to the four-tier scoring system (0–3) of de Freitas et al. [20] and occasional loss of outer hair cells (OHCs) and supporting cells in the organ of Corti were consistently noted after evaluation across numerous sections. Although some sections appear relatively preserved, scoring across multiple sections and animals consistently yielded an average score of 2, which represents moderate damage (represented in Figure 1C). Atrophy and cytoplasmic vacuolization were observed in the marginal cells, while spiral ganglion cells (SGs) exhibited moderate nuclear degeneration and vacuolization. Degeneration of the tectorial membrane and supporting cells was also evident. In contrast, Group IV (lactoferrin) had a mean score of 0–1, indicating a relatively maintained normal cochlear architecture with only minimal alterations compared with the blank and negative control groups. Some apparent cell loss in this group may be attributable to sectioning artifacts rather than true degeneration, as cochlear sections are known to be prone to distortion during decalcification and microtomy (Figure 1).

**Figure 2:**
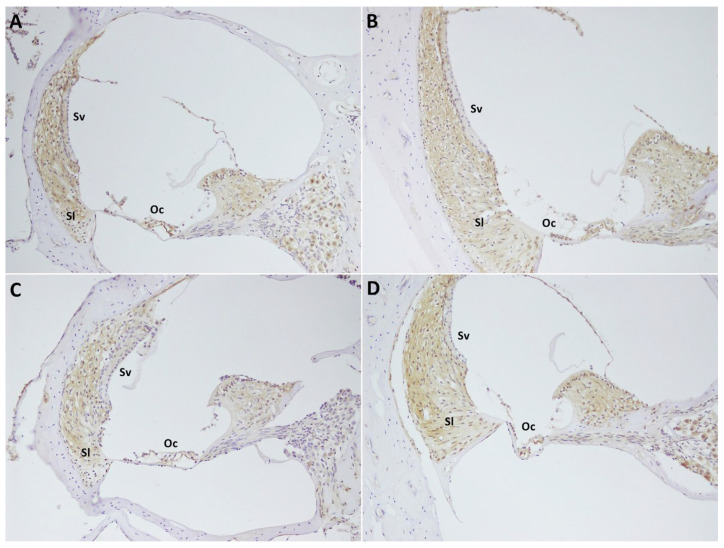
Vascular endothelial growth factor (VEGF) immunostaining of cochlear structures. (**A**) Group I, blank control; (**B**) Group II, 0.9% isotonic sodium chloride; (**C**) Group III, antiseptic solution (70% isopropyl alcohol + 2% chlorhexidine solution); (**D**) Group IV, lactoferrin. Oc: Organ of Corti. Sl: Spiral ligament. Sg: Spiral ganglion. Sv: Stria vascularis.

Immunohistochemical evaluation focused on the stria vascularis (SV), spiral ligament (SL), spiral ganglion cells (SGs), and organ of Corti (OC), which are major sites of VEGF expression in the rat cochlea. Staining intensity was categorized as weak, moderate, or strong [23]. Groups I, II, and IV demonstrated predominantly moderate to strong VEGF immunoreactivity, whereas Group III showed markedly reduced immunoreactivity, with weak to moderate staining (Table 5, Figure 2).

These histological and immunohistochemical observations strongly support the electrophysiological results, indicating that antiseptic exposure induces structural and molecular cochlear damage, while lactoferrin exerts a protective effect by preserving tissue integrity and VEGF expression.

The thickness of the SV (Group I = 11.02 ± 1.64 µm, Group II = 12.07 ± 1.56 µm, Group III = 15.75 ± 1.23 µm, Group IV = 10.69 ± 1.12 µm) was compared between the groups. The thickness in Group I, II, and IV was significantly smaller than Group III (*p* = 0.002, *p =* 0.005, *p =* 0.001, respectively).

## 4. Discussion

Harboring antimicrobial, anti-inflammatory, anticarcinogenic, and antioxidant features together in one, the multipower molecule lactoferrin serves as a potential future prospect for a wide range of diseases including otologic disorders [1,24]. In this regard, it is of utmost importance to know the molecule’s safety and potential harm beforehand for the inner ear, which comprises delicate sensory elements. This study revealed remarkable effects of lactoferrin on the inner ear electrophysiologically and histopathologically.

Pharmaceutical delivery to the inner ear can be achieved through numerous ways, with the intratympanic route being one of the most common and recognized methods for effective administration [25,26]. Chemicals can diffuse inside the cochlea through promontorial venous plexus, oval and round window, and dehiscent areas of the labyrinth [27]. The antiseptic solution of alcohol and chlorhexidine mixture (Wang X) used as the positive control in our study elicited the anticipated ototoxicity both electrophysiologically and histopatohologically through intratympanic injection, assuring the route of delivery in the experiment. It should be noted that more pronounced hearing loss, ranging from severe to profound, was induced at higher frequencies (16, 24, and 32 kHz), corresponding to the middle and basal turns of the rat cochlea. This finding aligns with the tonotopic organization of the human cochlea, suggesting a similar frequency-specific vulnerability in both species. The effects observed following the administration of the known ototoxic agent via the specified route confirm that the substance primarily reaches the basal turn through the mentioned pathways and exerts its initial toxic effect there.

Ensuring an atraumatic technique during intratympanic injection is crucial to minimize potential complications. After a clear vision of the tympanic membrane was provided through microscopic magnification with a suitable size ear speculum, a delicate thin needle was used to perform injections to prevent accidental injury of the external ear canal, perforation of the tympanic membrane, or disruption of the ossicular chain. These adverse outcomes can contribute to hearing loss with additional conductive components, independent of the administered agent. Modest increase in the hearing levels of the negative control group, where 0.9% isotonic sodium chloride solution was administered, was also seen in other intervention groups, indicating the mechanics of the procedure itself had no significant influence on the results.

Electrophysiological assessments play an essential role in revealing the hearing function of the inner ear and identifying compromised auditory performance. When the ABR test results in our study were evaluated, lactoferrin notably exhibited the same pattern with 0.9% isotonic sodium chloride solution at low frequencies (8, 16, and 24 kHz). Both groups exhibited a slight but significant hearing loss in the early phase compared to basal hearing levels; yet, this loss was self-limiting and showed no further progression. The resulting hearing loss followed by partial recovery on day 21 in the negative control group is likely attributable to mechanical stress and transient effects of intratympanic injection itself. The absence of a statistically significant difference between Group II and IV suggests that lactoferrin does not cause ototoxicity at low frequencies; in the meantime, it is unclear whether lactoferrin is otoprotective with its anti-inflammatory and regenerative effects in this improvement or the recovery is merely due to the natural healing process.

Evaluation of the electrophysiological results at higher frequencies, however, revealed a distinct pattern in the lactoferrin-administered group compared to those observed at lower frequencies. At 32 kHz, the hearing loss observed on day 7 persisted and further deteriorated by day 21, with the hearing threshold reaching nearly 55 dB. This resistant and progressive hearing impairment indicates that lactoferrin has no protective effect at higher frequencies as it remains insufficient in preventing functional loss. Moreover, considering that significant late-phase hearing loss was elicited, this outcome may be better described as an ototoxic effect rather than merely an indistinct protective response. This difference between the lower and higher frequency results may be attributed to the tonotopic organization of the cochlea and the diminished cytoprotective efficacy of lactoferrin in the basal region. The cochlea and its vascular structure show different characteristics between its basal and apical parts. The basal region of the cochlea, which is responsible for processing high-frequency sounds, is histopathologically more vulnerable to insults and tends to be affected earlier by vascular changes [28]. Some studies attribute this susceptibility to the basal turn’s reduced vascularization and weaker blood supply [29]. Additionally, the basal region of the cochlea is more susceptible to oxidative stress [30,31]. Lactoferrin, although primarily recognized for its antioxidant properties, can exhibit paradoxical effects due to alterations in its iron-binding capacity. Certain conditions such as the pH level of the milieu and the iron saturation status can alter lactoferrin’s ability to sequester iron, which can disrupt oxidative balance and potentially induce oxidative stress [32]. This pro-oxidant effect has been reported in some studies, where lactoferrin exacerbated oxidative stress in metabolically active cells such as virus-infected neural cells and cancer cells [33,34,35]. This phenomenon is particularly concerning in the inner ear, which harbors sensitive and highly metabolically active cells [36]. According to our results, it appears that lactoferrin’s protective vascular effects and antioxidant effects may have been insufficient to counteract the elevated oxidative stress in the basal region. Furthermore, lactoferrin itself may have contributed to oxidative stress by increasing reactive oxygen species (ROS) levels in this area, which may potentially differ in pH from the apical part due to the differences in vasculature. Moreover, the limited circulation in the basal turn may have hindered the effective clearance of these ROS, further exacerbating the oxidative stress and metabolic burden. This may explain the persistence and progression of hearing loss observed in this area.

Histopathological examination provided valuable insights into the potential effects of lactoferrin on inner ear structures. In our study, Group IV exhibited preserved morphological structure in OHC and sustentacular cells, with no significant cellular degeneration or vacuolization. Additionally, the structural architecture of the SV appeared intact as its thickness remained consistent with normal values, indicating that the overall cochlear morphology maintained normal histological characteristics. These findings suggest that the preserved inner ear structure in the lactoferrin-administered group may be attributed to lactoferrin’s potential tissue-protective and regenerative properties. Notably, the significant thickening of the SV observed in Group III was not present in Group IV, further suggesting that lactoferrin may exert a protective effect on the stria vascularis, potentially through its role in reducing inflammation and regulating vascular homeostasis in this context.

VEGF is a key biomarker in the inner ear that regulates the cochlear vascular system. It is naturally expressed at high levels in regions such as the stria vascularis, spiral ligament, organ of Corti, and spiral ganglion, where it plays a critical role in angiogenesis and revitalizing blood circulation [37]. Importantly, VEGF expression decreases when these structures are damaged, as reported in studies where tissue injury was associated with reduced VEGF levels, indicating vascular compromise and impaired cellular function [38]. In our study, VEGF immunoreactivity was observed at strong to moderate (+++/++) levels in the stria vascularis, spiral ligament, organ of Corti, and spiral ganglion regions in Group IV, as well as in Group I and II. This unchanged VEGF expression, maintained at normal levels, suggests that cochleas of these groups preserved their structural integrity and that lactoferrin exerted no harmful effects on inner ear microvasculature. The preservation of VEGF expression in the lactoferrin-administered group suggests the possibility that lactoferrin’s protective, anti-inflammatory, and tissue-repairing effects may also appear in the cochlea. Conversely, Group III exhibited weak to moderate (+/++) VEGF immunoreactivity, indicating potential tissue damage and vascular impairment. The reduction in VEGF expression in Group III aligns with the concept that VEGF downregulation is linked to structural injury and reduced cellular support in the presence of ototoxicity.

There are certain limitations in our study that should be noted. Firstly, while two experienced researchers conducted both the histopathological and immunohistochemical evaluations using light microscopy, this technique may not sufficiently reveal subtle morphological changes in OHCs in case of cochlear damage [39]. Electron microscopy, with its superior resolution, is more capable of detecting such fine structural alterations and could provide a more detailed assessment of damage in OHC and its associated ultrastructures [40]. Additionally, performing cochleaograms in future studies could provide a more comprehensive and quantitative assessment of hair cell loss by mapping the distribution and severity of OHC and inner hair cell (IHC) damage across different cochlear regions, offering valuable insights into the extent of cochlear injury [41]. Last but not least, conducting a more detailed histopathological and immunohistochemical analysis by evaluating the basal, middle, and apical regions of the cochlea separately would allow for a clearer interpretation of the results and facilitate a more precise correlation with the ABR findings. Alternatively, future studies should also utilize cochlear surface preparation techniques to more accurately demonstrate hair cell loss across different turns and to achieve a better correlation with functional outcomes.

Secondly, although we utilized ABR testing to objectively assess ototoxicity, hearing thresholds in our study were determined using the descending–ascending method with 10 dB step increments and decrements. While this method is commonly used in auditory research, its reliance on identifying the point where responses disappear and reappear may inherently involve some degree of subjectivity during wave analysis. Thus, combining other electrophysiological measurements such as otoacoustic emissions (OAE), auditory steady-state responses (ASSR), and electrocochleography (ECoG) can enhance sensitivity in evaluating cochlear function [42].

Although the matched-pair design increased sensitivity, the relatively small sample size limits the generalizability of our findings. The post hoc power analysis showed sufficient statistical power for operation time and pain scores, but relatively low power for complication and necrosis rates, indicating that these results should be interpreted with caution. Therefore, future studies with larger populations are warranted to validate these findings.

Another important limitation is the potential influence of environmental noise and device-related noise-reduction features, which may act as confounding factors. Although the tests were conducted in a quiet clinical environment, background noise was not objectively quantified with a sound level meter. These factors should be carefully considered when interpreting our results, as they may affect reproducibility and comparability across different settings and hardware configurations.

Lastly, we determined the dosing regimen for lactoferrin administration based on existing ototoxicity studies due to the absence of standardized protocols for our study molecule [43,44]. Conducting controlled animal experiments incorporating different dosing regimens with different application intervals would allow for a more comprehensive evaluation of lactoferrin’s otoprotective or ototoxic potential [45]. Moreover, lactoferrin is a complex molecule with multiple forms that may exhibit altering biological properties. Variations in lactoferrin’s structure can arise due to differences in its source, such as being derived from humans or bovines, its iron saturation status, including forms such as apo-lactoferrin or holo-lactoferrin, and its glycosylation pattern, all of which can significantly influence its biological effects [46,47,48]. These structural differences may alter lactoferrin’s antioxidant capacity, anti-inflammatory effects, and cellular uptake mechanisms in the target tissue. In our study, we utilized recombinant human lactoferrin. The unique characteristics of the lactoferrin used in our study may have served as a key factor in shaping our results, and it is important to consider lactoferrin’s molecular variability when interpreting our findings and comparing them with previous research. Further studies may investigate the effects of different forms of lactoferrin on cochlear structures for a deeper understanding.

## 5. Conclusions

Lactoferrin, known for its antimicrobial, anti-inflammatory, and antioxidant properties, is a promising biomolecule with potential therapeutic applications in various medical fields, including otology. However, research investigating lactoferrin’s effects on the inner ear remains extremely limited. Our findings demonstrated that while lactoferrin appeared to be safe at lower frequencies based on ABR results, it exhibited a paradoxical ototoxic effect at higher frequencies, particularly at 32 kHz. Despite this, our histopathological and immunohistochemical results indicated that lactoferrin preserved inner ear morphology and had no evident adverse impact on cochlear microstructures, including the stria vascularis, spiral ganglion, outer hair cells, and supporting cells. These intriguing results suggest that lactoferrin’s potential harmful effects may selectively manifest in specific cochlear regions under particular conditions. Given these findings, it is crucial to approach lactoferrin’s use in the delicate inner ear with caution. Further investigations are warranted to clarify the conditions under which lactoferrin may act as an ototoxic agent. Future studies should evaluate the effects of different types of lactoferrin, varying dosing regimens, and administration protocols. Long-term follow-up will be essential to better clarify its potential otoprotective and ototoxic effects. Moreover, incorporating additional biochemical analyses, such as oxidative stress markers, may help elucidate the underlying mechanisms of lactoferrin’s effects, thereby strengthening future research and resolving existing uncertainties.

## Figures and Tables

**Table 1:** ABR thresholds (dB) at 8 kHz in all groups over time.

		Basal	7th Day	21st Day	*p*	p *
A (*n* = 16)	Mean ± SD Median Min–Max	22.8 ± 5.4 20 15–30	23.4 ± 6.7 20 15–40	23.4 ± 7.6 20 15–40	0.779	
B (*n* = 16)	Mean ± SD Median Min–Max	24.3 ± 5.1 25 15–30	36.5 ± 7 37.5 30–50	30.3 ± 6.4 30 20–40	**<0.001**	pre−7 ≤ **0.001** pre−21 = 0.155 7−21 = 0.155
C (*n* = 16)	Mean ± SD Median Min–Max	22.5 ± 5.7 20 15–30	53.4 ± 13.9 50 40–90	60.6 ± 13.8 60 40–100	**<0.001**	pre−7 = **0.002** pre−21 ≤ **0.001** 7−21 = 0.335
D (*n* = 16)	Mean ± SD Median Min–Max	24 ± 4.9 25 15–30	36.8 ± 3 35 30–40	35 ± 4 35 25–40	**<0.001**	pre−7 ≤ **0.001** pre−21 = **0.001** 7−21 = 1
p **		0.661	**<0.001**	**<0.001**		
p ***			A−B = **0.008** A−C ≤ **0.001** A−D = **0.004** B−C = **0.004** B−D = 1 C−D = **0.009**	A−B = 0.608 A−C ≤ **0.001** A−D = **0.013** B−C ≤ **0.001** B−D = 0.905 C−D = **0.005**		

A: Control, B: Saline, C: Antiseptic solution (70% isopropyl alcohol + 2% chlorhexidine), D: Lactoferrin. *p*: Within-group comparison (Friedman test, *p* < 0.05). * *p*: Post hoc test for within-group differences (Friedman, *p* < 0.05). ** *p*: Between-group comparison (Kruskal–Wallis test, *p* < 0.05). *** *p*: Post hoc test for between-group differences (Kruskal–Wallis, *p* < 0.05).

**Table 2:** ABR thresholds (dB) at 16 kHz in all groups over time.

		Basal	7th Day	21st Day	*p*	p *
A (*n* = 16)	Mean ± SD Median Min–Max	26.5 ± 3.9 27.5 20–30	25.9 ± 4.1 25 20–30	25.9 ± 4.1 25 20–30	0.135	
B (*n* = 16)	Mean ± SD Median Min–Max	30 ± 5.1 30 20–40	37.1 ± 7.7 40 30–60	33.7 ± 6.9 30 20–50	**0.009**	pre−7 = **0.040** pre−21 = 0.399 7–21 = 0.993
C (*n* = 16)	Mean ± SD Median Min–Max	28.7 ± 5.9 30 20–40	73.1 ± 21.2 70 50–110	75 ± 20.8 70 40–110	**<0.001**	pre−7 ≤ **0.001** pre−21 ≤ **0.001** 7–21 = 1
D (*n* = 16)	Mean ± SD Median Min–Max	26.8 ± 4.4 25 20–35	39 ± 5.2 40 30–50	36.8 ± 6.8 35 25–55	**<0.001**	pre−7 ≤ **0.001** pre−21 = **0.001** 7–21 = 1
p **		0.179	**<0.001**	**<0.001**		
p ***			A−B = **0.015** A−C ≤ **0.001** A−D = **0.001** B−C ≤ **0.001** B−D = 1 C−D = **0.005**	A−B = 0.096 A−C ≤ **0.001** A−D = **0.005** B−C ≤ **0.001** B−D = 1 C−D = **0.004**		

A: Control, B: Saline, C: Antiseptic solution (70% isopropyl alcohol + 2% chlorhexidine), D: Lactoferrin. *p*: Within-group comparison (Friedman test, *p* < 0.05). * *p*: Post hoc test for within-group differences (Friedman, *p* < 0.05). ** *p*: Between-group comparison (Kruskal–Wallis test, *p* < 0.05). *** *p*: Post hoc test for between-group differences (Kruskal–Wallis, *p* < 0.05).

**Table 3:** ABR thresholds (dB) at 24 kHz in all groups over time.

		Basal	7th Day	21st Day	*p*	p *
A (*n* = 16)	Mean ± SD Median Min–Max	15.3 ± 4.9 17.5 10–20	15 ± 4.8 15 10–20	15.3 ± 4.9 17.5 10–20	0.368	
B (*n* = 16)	Mean ± SD Median Min–Max	17.5 ± 5.7 20 10–30	29.6 ± 12.5 30 10–55	25 ± 10.9 20 10–40	**0.001**	pre−7 = **0.003** pre−21 = 0.126 7–21 = 0.648
C (*n* = 16)	Mean ± SD Median Min–Max	13.4 ± 4.3 10 10–20	63.4 ± 14.6 60 40–80	64.6 ± 13.3 60 30–80	**<0.001**	pre−7 ≤ **0.001** pre−21 ≤ **0.001** 7–21 = 1
D (*n* = 16)	Mean ± SD Median Min–Max	16.5 ± 3.9 15 10–25	29.6 ± 5.9 30 20–45	27.1 ± 5.7 25 20–40	**<0.001**	pre−7 ≤ **0.001** pre−21 = **0.001** 7–21 = 1
p **		0.127	**<0.001**	**<0.001**		
p ***			A−B = **0.037** A−C ≤ **0.01** A−D ≤ **0.01** B−C = **0.001** B−D = 1 C−D = 0.078	A−B = 0.136 A−C ≤ **0.01** A−D = 0.560 B−C = **0.001** B−D = 1 C−D = **0.002**		

A: Control, B: Saline, C: Antiseptic solution (70% isopropyl alcohol + 2% chlorhexidine), D: Lactoferrin. *p*: Within-group comparison (Friedman test, *p* < 0.05). * *p*: Post hoc test for within-group differences (Friedman, *p* < 0.05). ** *p*: Between-group comparison (Kruskal–Wallis test, *p* < 0.05). *** *p*: Post hoc test for between-group differences (Kruskal–Wallis, *p* < 0.05).

**Table 4:** ABR thresholds (dB) at 32 kHz in all groups over time.

		Basal	7th Day	21st Day	*p*	p *
A (*n* = 16)	Mean ± SD Median Min–Max	25.6 ± 5.4 27.5 20–35	25.3 ± 5.3 25 20–35	25 ± 5.1 25 20–35	0.223	
B (*n* = 16)	Mean ± SD Median Min–Max	24.3 ± 6 20 20–40	39.3 ± 11.3 40 25–70	35.6 ± 9.6 35 20–50	**<0.001**	pre−7 = **0.003** pre−21 = **0.014** 7–21 = 1
C (*n* = 16)	Mean ± SD Median Min–Max	23.1 ± 4.7 20 20–30	88.7 ± 10.2 87.5 70–100	90.3 ± 10.7 90 60–100	**<0.001**	pre−7 ≤ **0.001** pre−21 ≤ **0.001** 7–21 = 1
D (*n* = 16)	Mean ± SD Median Min–Max	25.9 ± 4.9 25 20–35	40 ± 7.3 40 30–55	54.6 ± 16.4 55 35–90	**<0.001**	pre−7 = **0.002** pre−21 ≤ **0.001** 7–21 = 0.231
p **		0.327	**<0.001**	**<0.001**		
p ***			A−B = **0.026** A−C ≤ **0.001** A−D = **0.006** B−C ≤ **0.001** B−D = 1 C−D = **0.002**	A−B = 0.346 A−C ≤ **0.001** A−D ≤ **0.001** B−C ≤ **0.001** B−D = 0.112 C−D = 0.077		

A: Control, B: Saline, C: Antiseptic solution (70% isopropyl alcohol + 2% chlorhexidine), D: Lactoferrin. *p*: Within-group comparison (Friedman test, *p* < 0.05). * *p*: Post hoc test for within-group differences (Friedman, *p* < 0.05). ** *p*: Between-group comparison (Kruskal–Wallis test, *p* < 0.05). *** *p*: Post hoc test for between-group differences (Kruskal–Wallis, *p* < 0.05).

**Table 5:** Immunoreactivity of vascular endothelial growth factor (VEGF).

	Stria Vascularis	Spiral Ligament	Organ of Corti
Group A	+++	+++	+++
Group B	++	+++	+++
Group C	+	+	++
Group D	+	++	++

A: Control, B: Saline, C: Antiseptic solution (70% isopropyl alcohol + 2% chlorhexidine), D: Lactoferrin. +: weak staining, ++: moderate staining, +++: strong staining.

## Data Availability

The data presented in this study are available on request from the corresponding author. The data are not publicly available as they contain raw experimental data not suitable for public sharing.
